# Epidemiological Study of COVID-19 Fatalities and Vaccine Uptake: Insight From a Public Health Database in Ontario, Canada

**DOI:** 10.7759/cureus.16160

**Published:** 2021-07-04

**Authors:** Gareth Leung, Ashish Verma

**Affiliations:** 1 Faculty of Medicine, University of Ottawa, Ottawa, CAN; 2 Nephrology, Brigham and Women’s Hospital/Massachusetts General Hospital, Boston, USA

**Keywords:** covid 19, vaccine science and policy, canada, elderly population, rural areas

## Abstract

Coronavirus disease (COVID-19) has reached millions of people worldwide and is responsible for millions of deaths around the world. Research on fatalities in rural communities remains limited. In addition, the scientific literature has not yet reported on the distribution of vaccines in Canada and compared the findings to the age distribution of COVID-19 fatalities in Canada to see whether the vaccines have been distributed to the highest age category populations. This research article used data from the Government of Ontario and Statistics Canada to analyze the number of cases, fatalities, case fatality rates (CFRs) by demographic factors, such as age, gender, urban-rural status, and compared the findings to national vaccination rates by age. As of June 11, 2021, this study found that among the 528,819 cases among 14.8 million people. Among this population, there were 8875 fatalities in Ontario with 82.208% (n=7296) of fatalities occurred in people over 70 years, and 93.183% in people over 60 years (n=8,270). Additionally, the odds ratio of a fatal event was 9,652 times higher in people over 90 years (95% CI: 4418, 31124, p<0.001) as compared with less than 20 years. Men had a higher number of fatalities (n=4,490, CFR=1.721%) compared with women (n=4,385, CFR=1.692%), and a higher odd of fatal events only when adjusted for age and gender (OR=1.66, 95% CI: 1.57, 1.74, p<0.001). Urban areas had 92.034% of fatalities (n=8,168) and had a CFR of 1.632%. In contrast, rural areas comprised 4.451% of total fatalities (n=395) and had the highest CFR (2.267%). The unadjusted odds of a fatality were 1.41 (95% CI: 1.27, 1.56) in rural areas compared with urban areas. Across Canada as of May 29, 2021, people over 80 years old received 1,530,318 vaccines with 91.98% of this population age group receiving at least one and 457,664 being fully vaccinated (27.51%). In Ontario, as the number of people with at least one vaccine increased for people over 90 years, the number of fatalities was reduced from about 8 per day prior to vaccines to approximately two per day. Furthermore, once the vaccination rates exceeded 75% in ages 60 years and over 50% in the younger age groups, the number of fatalities per day among all age groups was approximately one per day. In summary, age was found to be a significant factor for COVID-19 mortality in Ontario and vaccine uptake in Ontario was followed by decreases in COVID-19 mortality.

## Introduction

As of June 7, 2021, coronavirus disease (COVID-19) has reached over 173 million cases and over 3.7 million deaths globally [1]. Vaccinations represent a vital strategy to control the spread of the COVID-19 pandemic. With the availability of many COVID-19 vaccines, there is hope that the pandemic will be brought to an end [2]. Given the limited vaccines, there has been general scientific consensus that people in the over 65 age category, as well as those with underlying medical conditions, should be provided high priority [3-4]. At this time, however, there is a high degree of inequity regarding vaccine distribution, and many countries with vulnerable elderly populations have not yet received a vaccine [2].

Though the worldwide pandemic has brought a significant emphasis on studying COVID-19, research on rural populations remains limited [5]. According to one study, rural populations are being overlooked in the COVID-19 research literature, despite facing unique economic and health challenges [5]. According to the Centers for Disease Control, rural communities in the United States tend to have higher levels of hypertension, cigarette smoking, and obesity compared with urban areas [6]. However, research by Souch et al. found that populations with those previously mentioned cardiovascular risk factors had a lower COVID-19 test rate per 100,000 people [7]. Together, these findings suggest that rural areas may have lower access to COVID-19 testing facilities, but research on this topic has yet to be comprehensively studied.

The vaccinations began on December 15, 2020 [8], and the aim of this study was to retrospectively assess which populations have been most at risk of contracting COVID-19. As well, this study aimed to determine whether the vaccine distribution strategy reached the vulnerable populations and whether vaccine use was correlated to decreases in testing positive for COVID-19. Other outcomes assessed in this study include the distribution of fatalities by gender and rural-urban status. To our knowledge, there have been no prior large-scale studies in Canada reporting on the case fatality rates, rural-urban status, and vaccine distributions by age.

## Materials and methods

Due to data availability, data from Canada’s most populated province, Ontario, was used to calculate COVID-19 statistics from January 1, 2020, to June 11, 2021 [9]. Data were summarized by gender, age, rural/urban status, and location of the reporting public health unit [9]. A multivariable regression model was used to calculate odds ratios of fatalities by age, adjusted for gender, and the interaction term between age and gender was assessed. Case fatality rates by age group were calculated by dividing the number of fatalities in each age group by the total number of resolved COVID-19 cases with a known fatal or non-fatal outcome. The percent of positive cases during a seven-day period was obtained from the Government of Ontario website and analyzed by age group [10].

To classify cases as urban, rural, or mixed urban-rural areas, the public health unit that reported the COVID-19 case was used. Regions were classified by rurality based on Statistics Canada’s peer groups used to categorize health regions [11]. This classification scheme divides the province’s cities into regions based on variables such as population density, demographic factors, and living conditions [11]. In this study, urban regions were defined by those with a high population density, as designated by Statistics Canada’s regions A, B, G, and H. Rural areas were regions with low population densities, classified as D and E, while region C was classified as mixed rural-urban areas. The chi-square test was used to determine whether there was any significant difference in case fatality rates for those in rural vs. urban areas. Analyses were conducted in R 4.0.5 (R Core Team, Vienna, Austria) [12].

Data from Canadian vaccination databases were retrieved from December 19, 2020, to May 29, 2021, to compare vaccine distribution by age group [13]. Male and female vaccination rates were combined by age group to determine the total number and percentage of vaccines given in each age strata.

## Results

As of June 11, 2021, the study found that among 538,651 COVID-19 cases in Ontario, there were 538,551 people with complete data for age. Out of the 14,789,778 people in Ontario from April to June 2021, this represents 3.642% of the population having been positive for COVID-19 during the pandemic [14]. After excluding unresolved cases and people without sufficient identifiable information on age and gender, 528,819 people with either non-fatal cases or fatal outcomes were included for analysis.

Results by age and sex

Percent of Positive COVID-19 Cases by Age Over a Seven-Day Period

**Figure 1:**
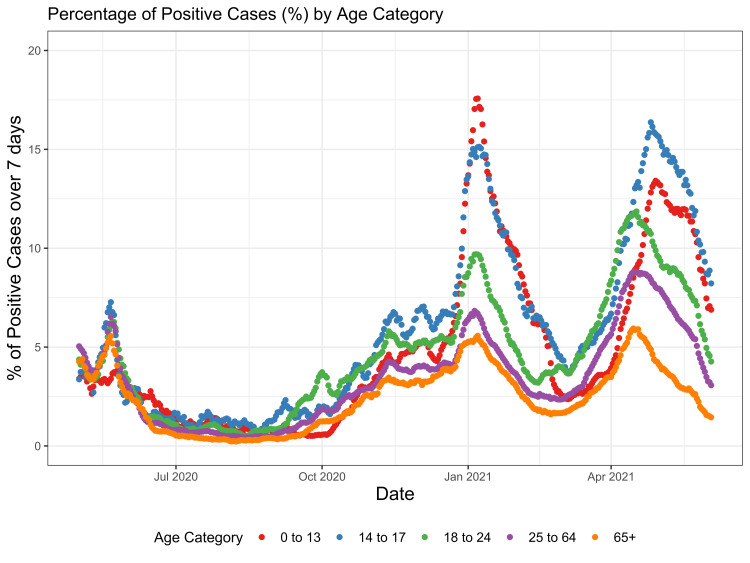
Percentage of Positive Cases (%) by Age Category

In the earlier phase of the pandemic near March 2020, the percentage of people with a positive COVID-19 test was relatively similar across age groups (Figure 1). At that time, the proportion of positive cases was lowest in people ages zero to 13 and higher among people over age 25; yet the rates were relatively similar. However, as the pandemic progressed and peaked near January 2021 and May 2021, the differences between age groups became more apparent. As seen in Figure 1, there was a positive association between younger age and a higher likelihood of percentage positive, as shown by the younger age strata having a higher positive test rate.

COVID-19 fatalities

Among the 8,875 fatalities, the overall case fatality rate was 1.678% (Table 1). Fatalities were highest in people over 90 years (n=2,407 deaths, CFR=28.468%) and lowest in those <20 years (n=4, CFR=0.005%) (Table 1).

**Table 1:** Fatalities and Case Fatality Rates

Age Group	Fatalities	Non-Fatal Cases	Case Fatality Rate (%)
<20	4	84076	0.005
20s	24	111731	0.021
30s	45	86040	0.052
40s	118	76079	0.155
50s	414	75065	0.548
60s	974	46127	2.068
70s	1812	21738	7.694
80s	3077	13040	19.092
90+	2407	6048	28.468
Total	8875	519944	1.678

Compared with cases <20 years, the odds of a fatal event in people 90+ years were OR=9,652 (95% CI: 4418, 31124, p<0.001), 80 to 89 years OR=5,328 (95% CI: 2291, 17176, p<0.001), and 70 to 79 years, OR=1777 (95% CI: 764, 5729, p<0.001) (Table 2). People over 70 years comprised 82.208% of deaths from COVID-19 (n=7296) and people over 60 years comprised 93.183% of deaths (n=8,270).

**Table 2:** Results from the Multivariable Logistic Regression

	Odds Ratio	95% CI	p-value
Gender			
Female	—	—	
Male	1.66	1.58, 1.74	<0.001
Age Group			
<20	—	—	
20s	4.49	1.73, 15.3	0.005
30s	11.0	4.48, 36.6	<0.001
40s	33.2	14.0, 108	<0.001
50s	117	50.2, 379	<0.001
60s	443	190, 1,431	<0.001
70s	1,777	764, 5,729	<0.001
80s	5,328	2,291, 17,176	<0.001
90+	9,652	4,148, 31,124	<0.001

**Figure 2:**
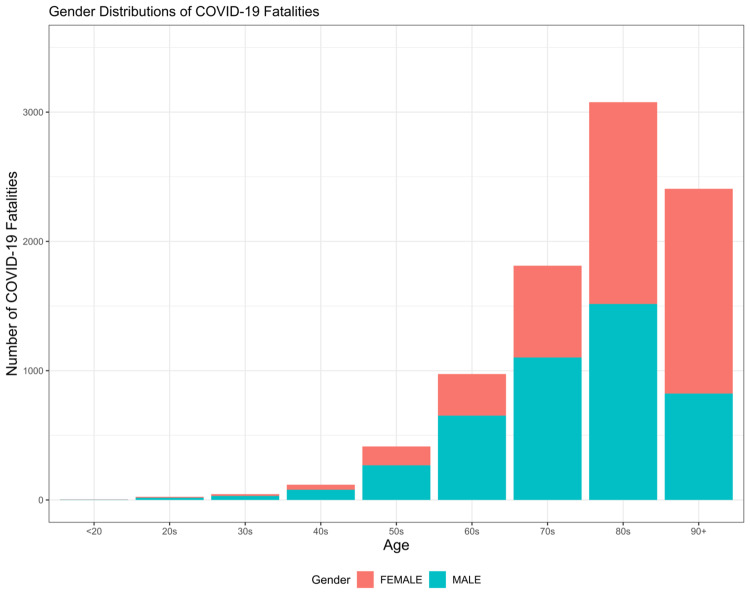
Gender Distribution of COVID-19 Fatalities

There were 260,784 cases among men and 259,160 cases among women. Of the total fatalities, men had a higher number of fatalities (n=4,490) as compared with women (n=4,385). This was equivalent to a CFR of 1.721% for men and 1.692% for women. By gender, the logistic regression adjusting for gender and age found that there was a statistically significant difference between gender and the odds of COVID-19 fatality (OR=1.66, 95% CI: 1.57, 1.74, p<0.001). As shown in Figure 2, there were more male mortalities in the <80 years while more female mortality >90 years. The logistic regression adjusting for the interaction term between age and the gender of COVID-19 fatality found was not statistically significant.

**Figure 3:**
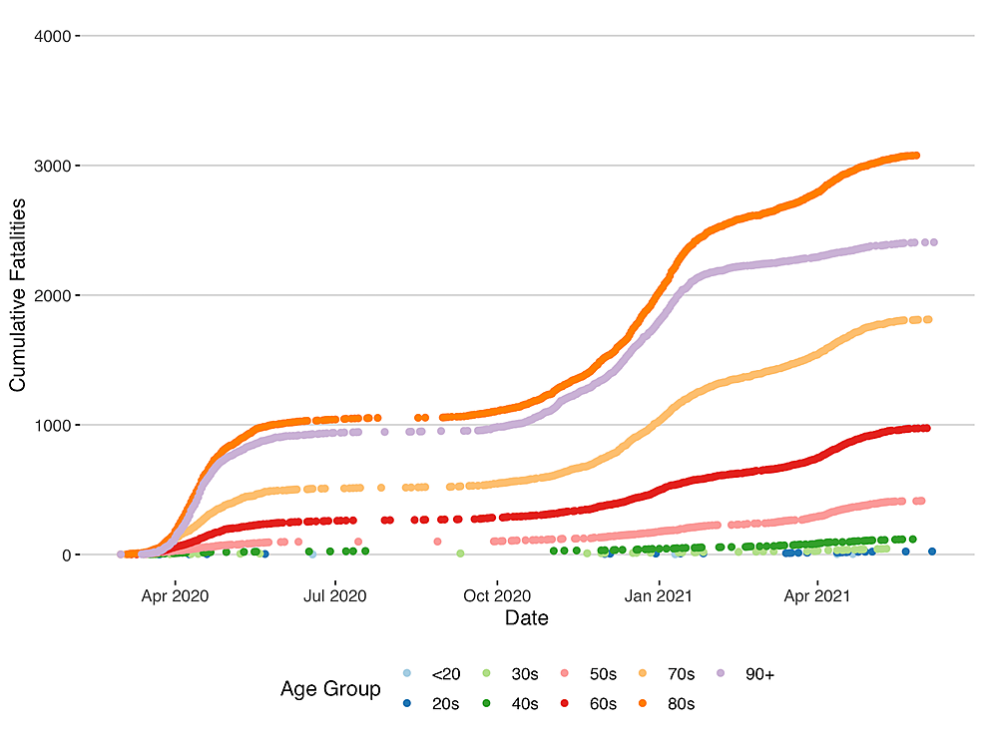
Cumulative Fatalities by Age in Ontario

As shown in Figure 3, the highest number of cumulative fatalities was in people over 80 years, followed by 90 years, 70 years, and 60 years, respectively. Figures for people under the age of 40 were comparatively low.

Urban-rural status

**Figure 4:**
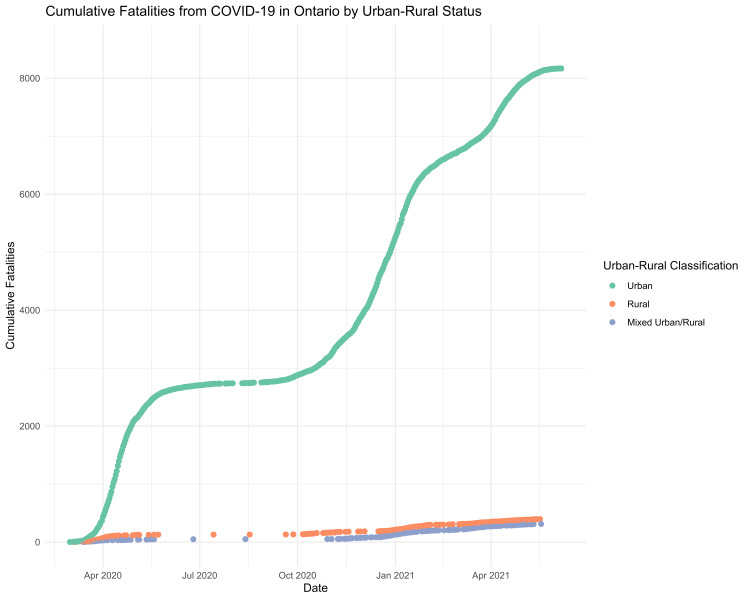
Cumulative Fatalities From COVID-19 by Urban-Rural Status

Urban areas had the majority of cases (n=492,222 cases), followed by mixed rural-urban regions (n=19,571 cases), and lastly rural areas (n=17,026 cases) (Figure 4).

Of the 8875 total fatalities in Ontario, 92.034% (n=8,168) occurred in urban areas, 4.451% (n=395) in rural areas, and 3.515% (n=312) occurring in mixed rural-urban areas. The case fatality rate was lowest amongst mixed rural-urban areas (1.569%), followed by urban areas (1.632%), and highest in rural areas (2.267%). The unadjusted odds ratio of a COVID-19 fatality in rural vs. urban areas was 1.41 (95% CI: 1.27, 1.56). Pearson’s chi-square test found that there was a statistically significant difference between rural-urban status and fatalities (chi-square = 42.666, df = 2, p-value = 5.434 e-07).

**Figure 5:**
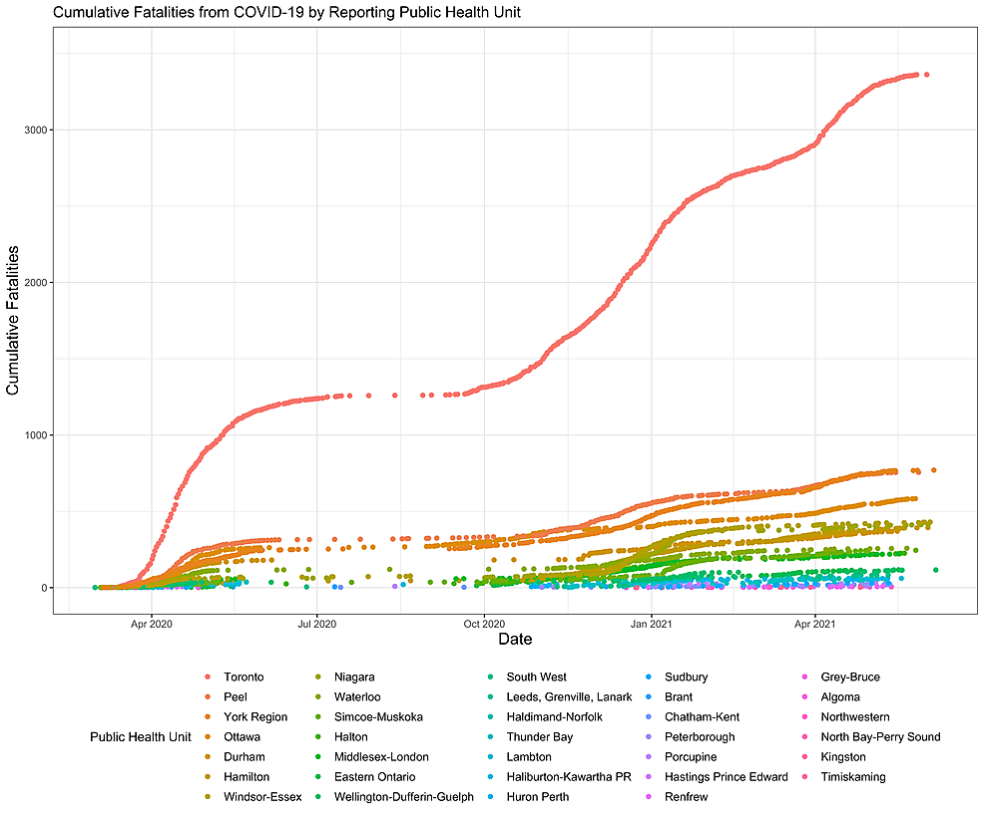
Cumulative Fatalities From COVID-19 by Reporting Public Health Unit

Most of the fatalities were reported by the Toronto public health unit (n=3362), followed by York (n=770), and Peel (n=756) (Figure 5). The lowest number of fatalities occurred in Kingston (n=3) and Timiskaming (n=3) (Figure 5).

Vaccine data

Across Canada, vaccines were distributed proportionally in the population based on their age groups. As of May 29, 2021, people over 80 years old received 1,530,318 vaccines with 91.98% of this population age group receiving at least one and 457,664 being fully vaccinated (27.51%) (see Table 3). Likewise, people over there were 2,748,537 people over 70 years old who received at least one vaccine, comprising 91.47% of this population, and 298,330 people were fully vaccinated (9.93%). In contrast, people in the lowest age categories had the lowest vaccination rates, both in terms of partial and full vaccinations (Table 3).

**Table 3:** Vaccinations as of May 29, 2021, in Canada Data source: Public Health Agency of Canada

Age Group	Partially Vaccinated N, (%)	Fully Vaccinated N, (%)
0-17	556897 (7.68%)	5982 (0.08%)
18-29	2684627 (44.62%)	216367 (3.6%)
30-39	2927373 (55.31%)	265165 (5.01%)
40-49	3214705 (66.22%)	284397 (5.86%)
50-59	3840488 (73.93%)	320783 (6.18%)
60-69	4027728 (85.2%)	298601 (6.32%)
70-79	2748537 (91.47%)	298330 (9.93%)
80+	1530318 (91.98%)	457664 (27.51%)
All	21551460 (56.7%)	2161780 (5.68%)

Temporal relationship between vaccine distribution and fatalities in Ontario

**Figure 6:**
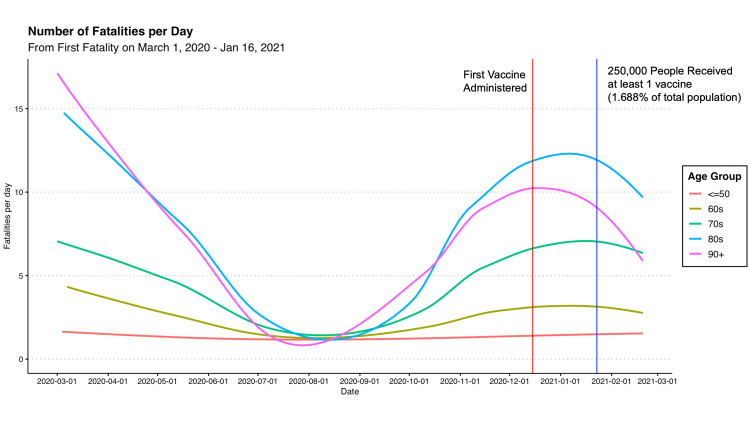
Number of Fatalities Per Day from COVID-19: From First Recorded Fatality Until Vaccine Data by Age Available

According to results from the Government of Ontario database, the first recorded case of COVID-19 in Ontario occurred on January 1, 2020 [9]. Three months later, the first recorded case of COVID-19 with a fatal outcome was recorded on March 1, 2020. As shown in Figure 6 below, the number of fatalities from COVID-19 prior to vaccinations remained high, reaching an average of 10 fatalities per day during peak waves in March 2020 and January 2021 in the 80 and 90-year-old age groups. Since the first COVID-19 vaccine administered on December 15, 2020, the distribution of fatalities per day plateaued and decreased in age groups 60 and older [15].

**Figure 7:**
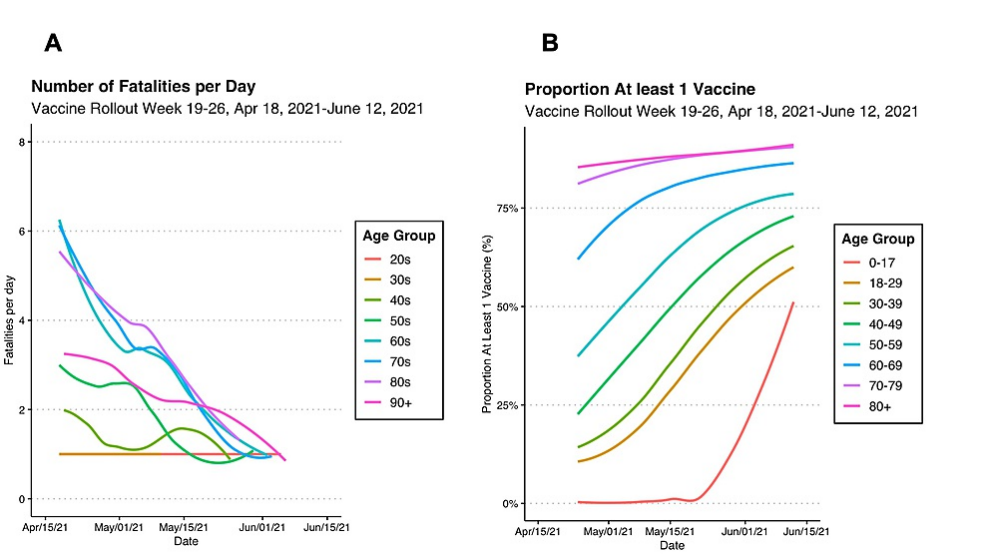
COVID-19 Fatalities in Ontario by Age Compared With Vaccine Distribution Weeks 19-26, Apr 18, 2021, to June 12, 2021

**Figure 8:**
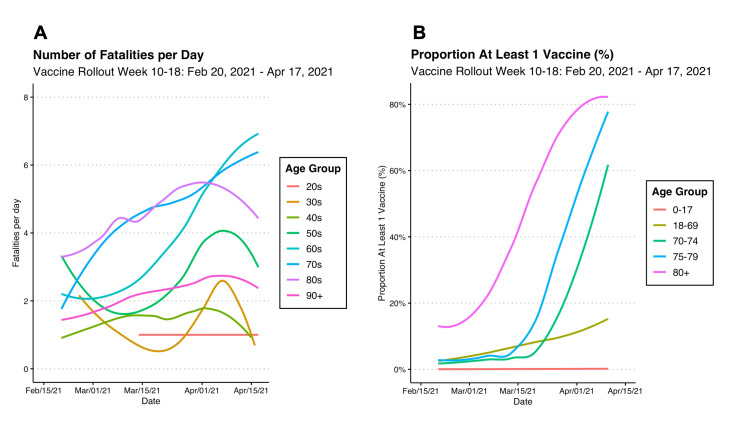
COVID-19 Fatalities by Age in Ontario Compared With Vaccine Distribution Weeks 10-18, Feb 20, 2021, to Apr 17, 2021

Due to limited data availability on vaccine distribution, only vaccines from February 20, 2021, were available with age-stratified data. At that time, the first 387,269 vaccine doses had been administered, and this was 10 weeks since the first dose on December 15, 2020. Nevertheless, the data shown in Figure 7 was relatively early in the province's distribution strategy, as only 15% of people over 80 had at least one vaccine and less than 5% in other age groups (shown in Figure 8, panel B).

As more COVID-19 vaccines were administered, the number of fatalities was correlated with lower levels of fatalities per day. As shown in Figure 7, panel A below, the number of fatalities was approximately 4 per day, which was lower than in previous months. In people over 90, who received the first vaccines, the number of fatalities was no longer high and was approximately 2 per day (Figure 8, panel A). As shown in Figure 8, panel B, as people over 80 years exceeded 80% vaccination, there appeared to be a decrease in the trajectory of fatalities shown in Figure 8, panel A.

On April 18, 2021, 19 weeks into the vaccine distribution strategy, there appeared to be an evident decrease in the number of COVID-19 fatalities (Figure 7, panel A). As of June 12, 2021, the number of fatalities across all age groups was approximately one per day (Figure 7, panel A). As shown in Figure 7, panel B, this was correlated to a rise of COVID-19 vaccinations above 75% in ages over 60 and above 50% in all other age groups.

## Discussion

The current study provides evidence that Canada's vaccination has successfully prioritized older age groups in the distribution strategy. This is essential because people <20 years had 7800 times higher odds of a fatality as compared to >80 years in Ontario. Since the start of the pandemic, 93% of COVID-19 fatalities occurred in people over 60 years, and 82% occurred in people over 60 years.

Our findings with regards to age agree with findings from the United States Centers for Disease Control (CDC), which found people over age 85 had an 8700 higher odds of fatal events compared to the reference group of age 5-17 [16]. The CDC also found that among people age 75 to 85, they had 3200 times higher odds of a fatal event compared to age 5-17 years; ages 65 to 75 had a 1300 times odds; and ages 50 to 65 years had a 440 times the odds [16]. Another study of 22 countries found that people over age 70 years comprised at least 80% of fatalities in most countries [17].

Moreover, our analysis showed that there was a temporal relationship between vaccinations and lower rates of fatal COVID-19 events among older age groups. Prior to vaccinations, people over age 80 and 90 consistently had much higher fatalities from COVID-19 at around 8 to 10 fatalities per day during the peaks of the pandemic in March 2020 and January 2021 (Figure 6). However, since the start of vaccinations, the fatalities decreased to around three to five per day (Figure 8, panel A), respectively, putting them lower than ages 60 and 70 who did not yet receive the vaccines (Figure 8, panel B). Additionally, as more than 80% of people became vaccinated (Figure 7, panel B), the number of fatalities in these high-risk age groups and other age groups decreased to about one per day (Figure 7, panel A).

Upon additional analysis, Canada's strategy as a whole reflects a similar approach to Ontario where more data were available for analysis. As reported by Statistics Canada, vaccines were given to >90% of people over 70 years and >85% of people over 60 years as of June 11, 2021 [13]. In Canada, even though only 5.68% have been fully vaccinated, the majority of the highest age groups have been vaccinated. This shows that in limited-resource settings, a targeted approach can be used to protect a large proportion of vulnerable people.

From a global perspective, this research also raises questions about health protection for the broader global community as advocated by the World Health Organization [18]. When many of the world’s population is still unvaccinated, ethical questions about whether it is fair to vaccinate younger people in more wealthy nations while the elderly and most vulnerable in less wealthy nations are still at a higher risk of fatal outcomes [4,19]. With many people in wealthy nations looking to travel, some researchers have stated that vaccine equity should be the priority over vaccine passports [20]. They have advocated that the vaccine passports may only increase global vaccine inequities in favor of people in high-income countries [20]. Estimates have suggested that 80% of people in low-income regions will not be vaccinated in 2021 and that the COVID-19 Vaccines Global Access (COVAX) program will aim to vaccinate only 20% of its target countries by 2021 [4]. Evidence suggests that this may be due to a combination of factors, including a lack of technological resources to effectively distribute and schedule vaccinations [21].

Research from mathematical modeling studies has suggested that vaccinating the most vulnerable saves not only the greatest number of lives but also protects against the greatest number of years lost due to COVID-19 [22]. The results of another study on COVID-19 in over 600,000 cases across five countries found that mortality was less than 1% in those 50 years old, and highest in those over age 80 years. Together, these findings suggest that given the limited access to vaccines, people especially those over age 70 years and 80 years should be prioritized when providing vaccines to low-income countries.

Results of the urban-rural analysis suggest that most of the COVID-19 cases and fatalities occurred in urban areas (n=492,222 cases, n=8,168 fatalities) rather than rural areas (n=17,026 cases, n=395 fatalities). However, when adjusting for population size, the CFRs were higher in rural areas (CFR=2.267% in rural vs. CFR=1.632% in urban regions, p<0.001). A study in the United States examining COVID-19 fatalities from February 2020 to June 2020 found that COVID-19 fatalities were highest among urban compared with rural areas [23]. Yet, the fatality rates in this study were also lower in rural counties (incidence rate ratio = 0.297, SE=0.022) as compared with urban areas [23]. These findings indicate that while most of the resources should be concentrated in urban areas to protect against the highest number of fatalities, further research regarding the higher CFRs found in this study requires further examination.

Evidence from Peters et al. suggests that rural areas may be at higher risk of COVID-19 fatalities because they tend to have fewer physicians, less access to healthcare services, and older populations [24]. Other evidence suggests that the higher CFR among rural areas may be due to the lack of testing in rural regions [7]. If there is a lack of testing, people with less severe COVID-19 symptoms would not be accounted for, making the disease appear more fatal in those seeking medical assistance. Future steps suggest that additional research is needed to determine how best to support rural areas.

Strengths of this research include the large dataset and comprehensive data collection system that allowed for the inclusion of more than a million confirmed cases of COVID-19. Furthermore, the database contained information on cases collected for more than a year and a half since the inception of the pandemic, allowing for a greater understanding of how the pandemic has changed over time. This study also examined the temporal relationship between vaccination rates and fatalities by age, showing that lower fatality rates from COVID-19 fatalities followed vaccine uptake. Additionally, the study included geographic data that allowed for the association of COVID-19 cases and rurality. This evidence showed that though urban areas had more fatalities, rural areas had the highest case fatality rates.

Limitations of this dataset are primarily related to the lack of data. Since there was no readily accessible data on cases and fatalities throughout Canada, only data from Ontario could be used to compute summary statistics on fatality rates. Moreover, the Ontario dataset did not contain data on comorbidities such as obesity, kidney disease, cardiovascular disease, and respiratory conditions that may be confounders regarding fatality rates [25]. Lastly, the dataset did not contain data on race or ethnicity, socio-economic status, and educational level, which limited the ability to draw conclusions regarding the findings of the research [26-27].

## Conclusions

In conclusion, this study further contributes to the literature by describing the temporal relationship between vaccine uptake among elderly people and lower daily fatalities from COVID-19. This study also calculated odds ratios between different age categories using a large amount of data to further emphasize the importance of protecting elderly people. The vaccination strategy in Ontario was in prioritized this group and the lower fatality rates were most evident when vaccination rates exceeded 80% in people over 60 years, and over 50% in those other age groups. This research article also found that COVID-19 fatalities were largely distributed in urban areas, but rural areas had higher case-fatality rates, suggesting further research is needed. During the peaks of the pandemic, people in the younger age categories were at higher risk of testing positive for COVID-19 while those older were more likely to experience fatal events.

The findings discussed in the article reflect the viewpoints of the authors only and not of their respective institutions.
